# Expression Profiles of ITGA8 and VANGL2 Are Altered in Congenital Anomalies of the Kidney and Urinary Tract (CAKUT)

**DOI:** 10.3390/molecules29143294

**Published:** 2024-07-12

**Authors:** Nikola Pavlović, Nela Kelam, Anita Racetin, Natalija Filipović, Zenon Pogorelić, Ivana Kuzmić Prusac, Katarina Vukojević

**Affiliations:** 1 Department of Anatomy, Histology and Embryology, School of Medicine, University of Split, 21000 Split, Croatia; nikolapavlovic326@gmail.com (N.P.); katarina.vukojevic@mefst.hr (K.V.); 2 Department of Pediatric Surgery, University Hospital of Split, 21000 Split, Croatia; 3 Department of Surgery, School of Medicine, University of Split, 21000 Split, Croatia; 4 Department of Pathology, University Hospital Centre Split, Spinciceva 1, 21000 Split, Croatia; 5 Department of Anatomy, School of Medicine, University of Mostar, 88000 Mostar, Bosnia and Herzegovina; 6 Center for Translational Research in Biomedicine, School of Medicine, University of Split, 21000 Split, Croatia

**Keywords:** ITGA8, VANGL2, CAKUT, congenital anomalies of the kidney and urinary tract

## Abstract

Kidney failures in infants are mostly caused by congenital anomalies of the kidney and urinary tract (CAKUT), which are among the most common congenital birth disorders worldwide when paired with cardiac abnormalities. People with CAKUT often have severe kidney failure as a result of a wide range of abnormalities that can occur alone or in conjunction with other syndromic disorders. In this study, we aimed to investigate the expression pattern of CAKUT candidate genes alpha-8 integrin (ITGA8) and Van Gogh-like 2 (VANGL2) in fetal tissues of healthy and CAKUT-affected kidneys using immunohistochemistry and immunofluorescence. We found that under CAKUT circumstances, the expressions of ITGA8 and VANGL2 are changed. Additionally, we showed that VANGL2 expression is constant during fetal aging, but ITGA8 expression varies. Moreover, compared to normal healthy kidneys (CTRL), ITGA8 is poorly expressed in duplex kidneys (DKs) and dysplastic kidneys (DYS), whereas VANGL2 is substantially expressed in dysplastic kidneys (DYS) and poorly expressed in hypoplastic kidneys (HYP). These results point to VANGL2 and ITGA8 as potential prognostic indicators for CAKUT malformations. Further research is necessary to explore the molecular mechanisms underlying this differential expression of ITGA8 and VANGL2.

## 1. Introduction

Congenital anomalies of the kidney and urinary tract (CAKUT), together with heart defects, stand as one of the most common birth defects worldwide and represent a primary contributor to kidney failure in children [1,2]. Individuals affected by CAKUT have a broad spectrum of malformations, which can appear independently or as components of syndromic conditions, often resulting in end-stage kidney failure [3,4,5]. Cystic kidney dysplasia stands as one of the most prevalent CAKUT malformations. Other phenotypes include kidney hypoplasia, horseshoe kidney, kidney agenesis, vesicoureteral reflux (VUR), megaureter, ectopic ureter, duplex collecting system, and posterior urethral valves [6,7].

During nephrogenesis, nephrons can be classified into five different morphological stages: renal vesicle (stage I), comma-shaped body, S-shaped body, capillary loop nephron (stage III), and maturing nephron (stage IV) [8,9]. The metanephros development, which forms the permanent kidney, is divided into four phases based on the ureteric bud’s branching pattern and its ability to induce nephron formation. Phase 1, occurring from the 5th to the 14th week of development, involves each tip of the branching ureteric bud, inducing one nephron formation. Phase 2 starts around the 15th week and ends between the 20th and 22nd week, during which the ureteric tree stops branching, and each tip induces the formation of multiple nephrons that attach to it. Phase 3, lasting until the 36th week of development, sees each tip inducing several nephrons, which attach separately to the collecting duct. Phase 4, from the 36th week of development into adulthood, marks the cessation of new nephron formation as existing ones mature [9,10]. Various internal and external factors can disrupt nephrogenesis, leading to congenital anomalies of the kidney and urinary tract [11,12].

The alpha-8 integrin (ITGA8) is an integrin chain strongly expressed in mesenchymal and neuronal cells [13], and it has been demonstrated that ITGA8 stimulates neurite outgrowth, cell attachment, and spreading in neuronal cells [14]. ITGA8 plays crucial roles in kidney formation, especially during a pivotal developmental phase characterized by the reciprocal interplay between the epithelium of the ureteric bud, originating from the Wolffian duct, and the adjacent metanephric mesenchyme. This interaction is essential for triggering the outgrowth of the ureteric bud and initiating the differentiation of nephrons from the metanephric mesenchyme [15,16]. Humbert et al. demonstrated that bilateral renal agenesis is caused by mutations in the ITGA8 gene and that the condition is, at least in certain circumstances, autosomal recessive [17].

Van Gogh-like 2 (VANGL2), a key protein of the non-canonical Wnt/Planar Cell Polarity (PCP) signaling pathway, plays crucial roles in various developmental processes, including polarized cellular migration, neurulation, cardiac development, kidney-branching morphogenesis, and the regulation of hematopoiesis and organogenesis [18,19]. Regarding its physical structure, VANGL2 is a cell membrane protein featuring four consecutive transmembrane domains within its N-terminal half [20]. Derish et al. demonstrated that VANGL2 controls renal tubulogenesis during the embryonic stage. This observation may imply that congenital kidney abnormalities within the CAKUT spectrum may be caused by disruptions in PCP signaling [7].

Since both ITGA8 and VANGL2 play important roles in kidney development, it is of utmost importance to investigate their expression profiles during normal kidney organogenesis as well as during pathological outcomes. These genes have been chosen as potential CAKUT candidates. Therefore, our study focused on establishing the normal expression patterns of ITGA8 and VANGL2 during fetal development and comparing their expression in CAKUT-affected kidneys to potentially improve treatment outcomes through a precision medicine approach.

## 2. Results

Studies have shown that ITGA8 and VANGL2 play important roles during kidney development [13,19]. Therefore, we speculated that the expression pattern of ITGA8 and VANGL2 may be altered in CAKUT-affected fetal kidneys. We included 30 samples of human fetal kidneys (normal healthy kidneys vs. CAKUT-affected kidneys) at three developmental phases (Phase 2, Phase 3, and Phase 4) to verify this hypothesis. Phase 1 of kidney development was absent because of the extreme difficulty in obtaining samples at this early stage of development.

### 2.1. Hematoxylin and Eosin Staining (H&E) of Normal Healthy Kidneys and CAKUT-Affected Kidneys

**Figure 1:**
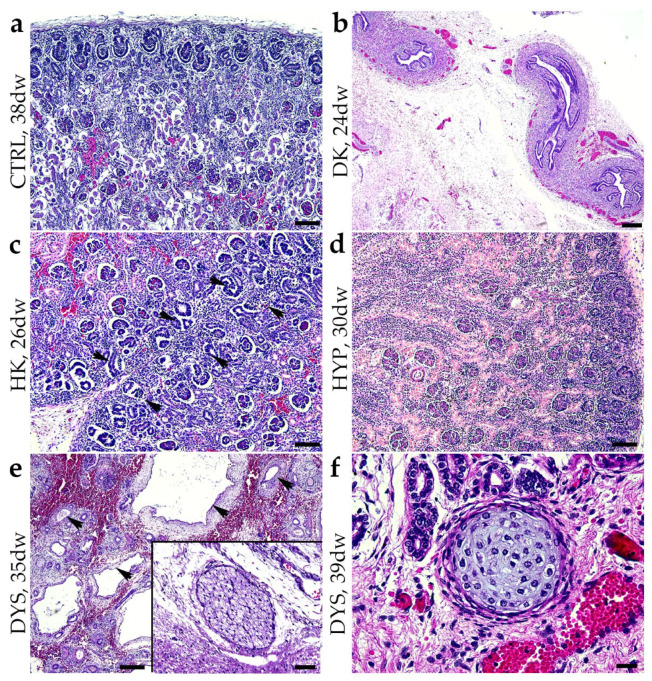
Hematoxylin and eosin (H&E) staining of normal healthy kidneys (CTRL) at the 38th developmental week (dw) (**a**) and CAKUT kidneys: duplex kidney (DK) at 24th dw (**b**), horseshoe kidney (HK) at 26th dw (**c**), hypoplastic kidney (HYP) at 30th dw (**d**), dysplastic kidney (DYS) at 35th dw (**e**), and 39th dw (**f**). Arrows show the most characteristic elements noticed for each phenotype. Images were taken at magnifications of 4× (**b**), 10× (**a**,**c**–**e**), and 40× ((**f**), insert). The scale bars are 20 µm ((**f**) insert), 100 µm (**a**,**c**–**e**), and 200 µm (**b**).

The normal healthy fetal kidneys (CTRL) display a well-formed nephrogenic area with cuboidal-shaped podocytes (Figure 1a). In a duplex kidney (DK), there are two ureters, but despite this, the renal tissue appears normal under microscopic examination (Figure 1b). A horseshoe kidney (HK) shows two nephrogenic zones with a primitive band where glomeruli and tubules form (Figure 1c). A hypoplastic kidney (HYP) resembles a normal kidney but with a narrower nephrogenic zone and fewer nephrons (Figure 1d). Dysplastic kidneys (DYS) exhibit abnormal nephron and tubule development, with irregularly arranged components and cysts originating from primitive ducts (PD). These ducts vary in shape, with tall columnar cells surrounded by fibromuscular tissue, and there is evidence of lobar disorganization of the kidney (Figure 1e). Rudimentary ducts (RDs) are found within connective tissue clusters, along with unmyelinated nerve fibers and areas of well-developed cartilage (Figure 1f).

### 2.2. ITGA8 Expression Declines with Fetal Age in Normal Healthy Kidney

**Figure 2:**
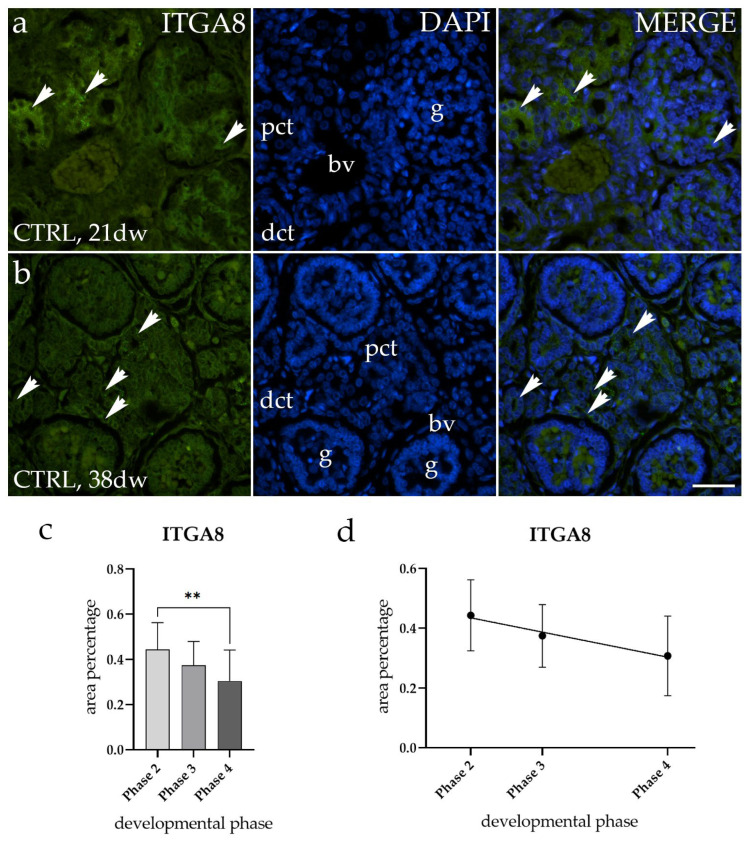
Immunofluorescence staining of normal healthy human fetal kidneys (CTRL) with the antibody for alpha-8 integrin (ITGA8) (**a**,**b**). Arrows show the expression pattern of ITGA8 in glomeruli (g), proximal convoluted tubules (pct), and distal convoluted tubules (dct) as indicated on the 4′,6-diamidino-2-phenylindole (DAPI) image. Immunoexpression of ITGA8, DAPI staining, and merged ITGA8 and DAPI in CTRL at the 21st (**a**) and 38th developmental week (dw) (**b**). Images were taken at a magnification of 40×. The scale bar is 100 µm for all images. The area percentages of ITGA8-positive cells in normal healthy fetal kidney tissues during developmental phases 2, 3, and 4 (**c**). Data are presented as the mean ± SD (vertical line) and analyzed using an ordinary one-way ANOVA test followed by Tukey’s multiple comparison test. Significant differences are indicated by ** *p* < 0.01. At each developmental phase, a minimum of ten representative pictures were assessed per observed region. The expression dynamics of ITGA8 (**d**) are shown using linear and nonlinear regression modeling of area percentages through developmental phases of CTRL fetal kidney tissues. Data are presented as the mean ± SD.

The spatiotemporal staining of ITGA8 in normal healthy fetal kidneys (CTRL) was detected as a diffuse green signal observed within the cytoplasm of the visceral layer of Bowman’s capsule (g), proximal convoluted tubules (pct), and distal convoluted tubules (dct). We observed significant differences in immunofluorescence signal intensity between the 21st developmental week (dw) (Phase 2) and the 38th developmental week (dw) (Phase 4) of CTRL kidneys (Figure 2a,b). A comparison of the area percentage of ITGA8-positive cells in normal healthy fetal kidneys revealed that the expression pattern of ITGA8 significantly decreases during fetal aging (*p* < 0.5, Figure 2c). No statistically significant difference was identified when a formal test for a linear trend among the observed developmental phases was conducted (R^2^ = 18.51%, β = −0.005989 ± 0.00379, Figure 2d).

### 2.3. ITGA8 Expression Is Altered in DK and DYS

**Figure 3:**
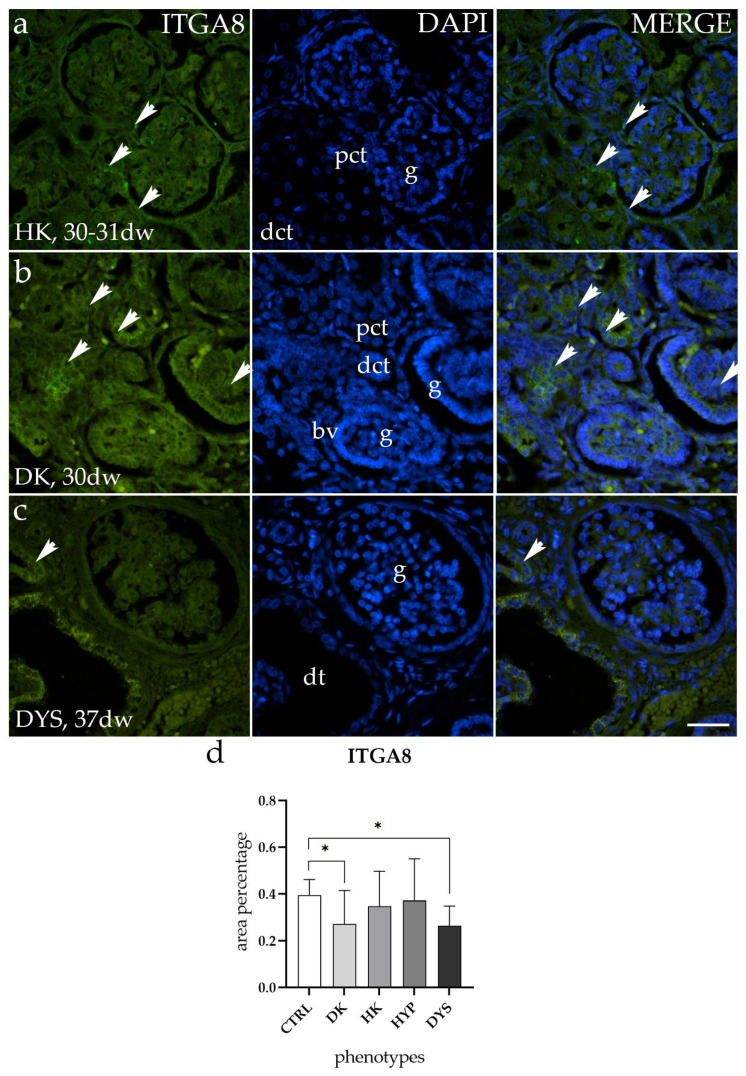
Immunofluorescence staining of human fetal kidneys with the antibody for alpha-8 integrin (ITGA8) (**a**–**c**). Arrows show the expression pattern of ITGA8 in glomeruli (g), proximal convoluted tubules (pct), distal convoluted tubules (dct), and dysplastic tubules (dt), as indicated in the 4′,6-diamidino-2-phenylindole (DAPI) image. Immunoexpression of ITGA8 and DAPI staining and merged ITGA8 and DAPI in horseshoe kidneys (HKs) at the 30th-31st developmental week (dw) (**a**), duplex kidney (DK) at the 30th dw (**b**), and dysplastic kidney (DYS) at the 37th dw (**c**). Images were taken at a magnification of 40×. The scale bar is 100 µm for all images. The area percentages of ITGA8-positive cells in the DK, HK, HYP, and DYS fetal kidney tissue (**d**). Data are presented as the mean ± SD (vertical line) and analyzed using an ordinary one-way ANOVA test followed by Tukey’s multiple comparison test. Significant differences were indicated by * *p* < 0.05. At each time point, at least ten representative pictures were assessed.

In both horseshoe kidneys (HKs) and hypoplastic kidneys (HYP), the ITGA8 staining patterns matched up in terms of both localization and intensity (Figure 3a). In duplex kidneys (DKs) and dysplastic kidneys (DYS), ITGA8 exhibits low expression levels, mostly within the glomerulus and the surrounding connective tissue (Figure 3b,c).

Comparing the area percentage of expression between normal healthy kidneys (CTRL) and those with congenital anomalies of the kidney and urinary tract (CAKUT), the results revealed that HK and HYP exhibited similar expression to CTRL, without significant differences, whereas a notable difference was observed in DK and DYS (*p* < 0.5, Figure 3d).

### 2.4. VANGL2 Shows Consistent Expression throughout All Stages of Normal Healthy Kidney Development

**Figure 4:**
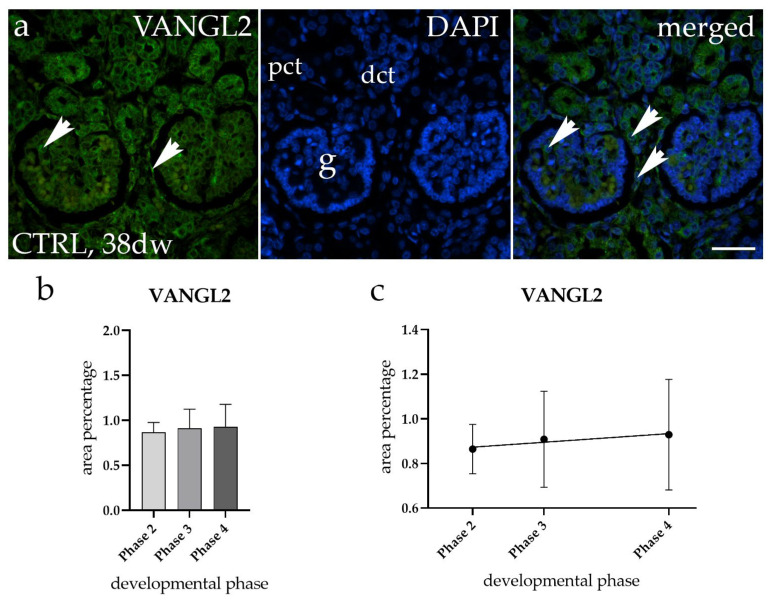
Immunofluorescence staining of normal healthy human fetal kidneys (CTRL) with the antibody for Van Gogh-like 2 (VANGL2) (**a**). Arrows show the expression pattern of VANGL2 in glomeruli (g), proximal convoluted tubules (pct), and distal convoluted tubules (dct), as indicated in the 4′,6-diamidino-2-phenylindole (DAPI) image. Immunoexpression of VANGL2, DAPI staining, and merged VANGL2 and DAPI images for CTRL at the 38th developmental week (dw) (**a**). Images were taken at a magnification of 40×. The scale bar is 100 µm for all images. The area percentages of VANGL2-positive cells in normal healthy fetal kidney tissues during developmental phases 2, 3, and 4 (**b**). Data are presented as the mean ± SD (vertical line) and analyzed using an ordinary one-way ANOVA test followed by Tukey’s multiple comparison test. No significant differences were observed. At each developmental phase, a minimum of ten representative pictures were assessed per observed region. The expression dynamics of VANGL2 (**c**) are shown using linear and nonlinear regression modeling of area percentages through developmental phases of CTRL fetal kidney tissues. Data are presented as the mean ± SD.

The spatiotemporal staining of VANGL2 in normal healthy fetal kidneys (CTRL) was detected as a diffuse green signal observed within the cytoplasm of the glomeruli (g), proximal convoluted tubules (pct), and distal convoluted tubules (dct). However, we did not observe any difference in expression pattern between different phases of the CTRL samples (Figure 4a).

Furthermore, a comparison of the area percentage of VANGL2-positive cells in normal healthy fetal kidneys revealed that the expression of VANGL2 remains constant during fetal aging (Figure 4b). No statistically significant difference was identified when a formal test for a linear trend among the observed developmental phases was conducted (R^2^ = 18.94%, β = 0.002764 ± 0.006336, Figure 4c).

### 2.5. VANGL2 Expression Is Altered in HYP and DYS

**Figure 5:**
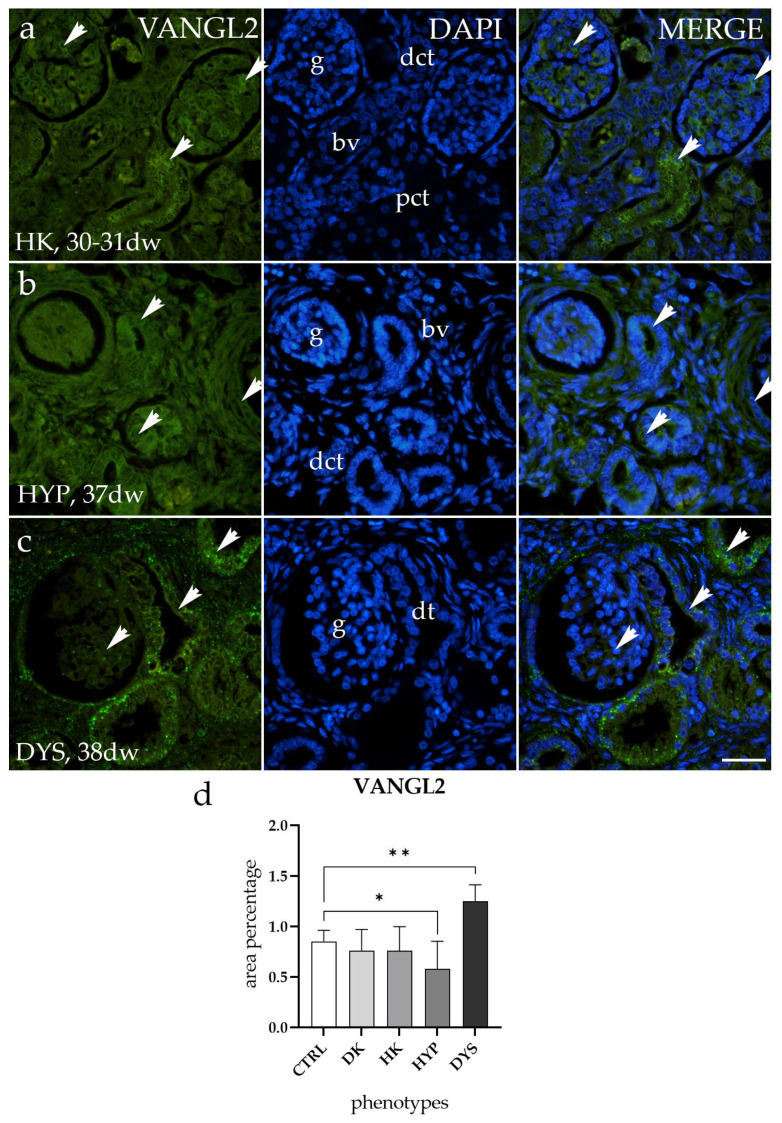
Immunofluorescence staining of human fetal kidneys with an antibody for Van Gogh-like 2 (VANGL2) (**a**–**c**). Arrows show the expression pattern of VANGL2 in glomeruli (g), proximal convoluted tubules (pct), distal convoluted tubules (dct), and dysplastic tubules (dt), as indicated in the 4′,6-diamidino-2-phenylindole (DAPI) image. Immunoexpression of VANGL2 and DAPI staining and merged VANGL2 and DAPI images in horseshoe kidneys (HKs) at the 30th-31st developmental week (dw) (**a**), hypoplastic kidney (HYP) at the 37th dw (**b**), and dysplastic kidney (DYS) at the 38th dw (**c**). Images were taken at a magnification of 40×. The scale bar is 100 µm for all images. The area percentages of VANGL2-positive cells in DK, HK, HYP, and DYS fetal kidney tissue (**d**). Data are presented as the mean ± SD (vertical line) and analyzed using an ordinary one-way ANOVA test followed by Tukey’s multiple comparison test. Significant differences were indicated by * *p* < 0.05 and ** *p* < 0.01. At each time point, at least ten representative pictures were assessed.

To evaluate whether the VANGL2 is differently expressed in CTRL and CAKUT-affected samples, we analyzed its staining pattern. We found that in both DK and HK, the VANGL2 staining patterns were similar in terms of both localization and intensity (Figure 5a). Results demonstrated lower levels of VANGL2 expression in HYP, observed as a diffuse green signal mostly within the glomerulus and surrounding connective tissue (Figure 5b), while VANGL2 exhibited higher expression levels in DYS, observed as a punctuated green signal mostly within dysplastic tubules (dt) (Figure 5c).

Additionally, when comparing the area percentage of expression between CTRL and those with CAKUT, the results revealed that DK and HK exhibited similar expression patterns to CTRL without significant differences, whereas a notable difference was observed in HYP and DYS (*p* < 0.5, Figure 5d).

## 3. Discussion

The etiology of CAKUT, both syndromic and nonsyndromic, has been linked to more than 40 chromosomal abnormalities and 50 genes [21]. In the present study, we found that ITGA8 and VANGL2 expressions are altered in CAKUT conditions. We also demonstrated that the expression of ITGA8 changes while the expression of VANGL2 remains constant during fetal aging. Additionally, ITGA8 is expressed at lower levels in duplex kidneys (DKs) and dysplastic kidneys (DYS) compared to normal healthy kidneys (CTRL), whereas VANGL2 is expressed at lower levels in hypoplastic kidneys (HYP) and at high levels in dysplastic kidneys (DYS). These findings suggest ITGA8 and VANGL2 as promising prognostic markers for CAKUT conditions.

ITGA8, a part of the cell adhesion receptors superfamily of integrins, is crucial for the development of the kidneys in humans. ITGA8 encodes the integrin α8 subunit, a cell surface protein mostly expressed on mesenchymal cells, such as kidney mesangial cells, fibroblasts, and vascular and visceral smooth muscle cells [22,23]. The integrin α8 subunit is essential for the proper development of the kidneys because it controls the mesenchymal cells’ epithelial conversion during nephrogenesis [24]. Renal fibrosis and congenital abnormalities of the kidney and urinary tract (CAKUT) are two renal conditions that may be influenced by dysregulation of ITGA8 expression or function [16]. Linton et al. demonstrated that the absence of ITGA8 results in renal agenesis and that nephronectin, a key factor in kidney development, is an essential ligand for ITGA8 during the initial events of kidney development [25]. They also reported that ITGA8 and its ligand nephronectin play a role in a pathway that regulates the expression of Gdnf, an essential growth factor. Our results showed a decrease in the expression of ITGA8 in DK and DYS, which could potentially impact normal epithelial–mesenchymal transition (EMT) and lead to the development of CAKUT. These results are consistent with previous studies indicating that defects in ITGA8 can result in congenital abnormalities of the kidney and urinary tract (CAKUT) and that mice lacking ITGA8 exhibit reduced kidney size or even kidney agenesis [16]. Also, we showed that the expression of ITGA8 decreases during normal healthy kidney development, which is expected since ITGA8 plays a role in the early stage of kidney development (especially during EMT). It has been previously shown that expanding the clinical and genotypic range of ITGA8 abnormalities reveals a high and unexpected degree of phenotypic heterogeneity in autosomal recessive disease [23]. Because ITGA8 expression varied in different CAKUT-affected kidneys, further research is necessary to explore the molecular mechanisms underlying this differential expression. Moreover, additional research is needed to examine the possible therapeutic uses of targeting ITGA8 in the context of CAKUT abnormalities.

VANGL2 is a crucial player in the establishment and maintenance of planar cell polarity (PCP), a conserved mechanism that is necessary for tissue organization and morphogenesis [18]. It has been reported that during the embryonic period, *VANGL2* controls renal tubulogenesis by regulating convergent extension (CE) and apical constriction (AC), and although disturbances in PCP signaling may lead to CAKUT phenotype, loss of *VANGL2* does not disturb mature renal architecture [26]. Mutations in PCP genes can result in dysplastic kidneys, among other multi-organ abnormalities [27]. Neural tube abnormalities have been linked to loss-of-function mutations in human VANGL2 genes, underscoring the significance of these genes in developmental processes [28]. According to Papakrivopoulou et al., in Xenopus embryos, *VANGL2* overexpression and knockdown result in significant bending or shortening of the body axis and abnormal neural tube closure [29]. Our data show that VANGL2 expression does not change during the development of normal, healthy kidneys, whereas VANGL2 expression changes in kidney hypoplasia and dysplasia, which is consistent with previous findings. Namely, kidney dysplasia in embryonic kidneys of the Lp mutant (a model with a mutation in the *VANGL2* gene, known for causing developmental abnormalities) has already been observed by Babyeva et al. [26], while Derish et al. [7] observed that embryonic *Vangl2^Δ/Δ^* kidneys displayed hypoplasia. Aggressive basal-type tumors were shown to have high expression levels of VANGL2, which is linked to poor prognosis [30]. Brzóska et al. [31] demonstrated that the interaction between *Celsr1* and *VANGL2* in the growth of the ureteric tree in the caudal compartment of the developing kidney is required for glomerular maturation. Further exploration of the VANGL2 protein could potentially lead to the development of targeted therapies for CAKUT abnormalities.

Our study has some limitations that must be considered. The primary limitation of our study lies in its small sample number for each type of CAKUT and the observational design. Observed samples include archived human fetal material that was formalin-fixed and paraffin-embedded; therefore, we were unable to use techniques like flow cytometry or Western blotting for quantitative protein expression analysis. Also, the lack of Phase 1 fetal kidney development samples limits the analysis and potential conclusions that can be drawn.

## 4. Materials and Methods

### 4.1. Human Fetal Kidney Tissue

Human kidney tissue was obtained following spontaneous pregnancy loss and eugenic abortions, which were marked by severe abnormalities, and was collected from the Department of Pathology at University Hospital Center Split. The tissue sampling adhered to the Declaration of Helsinki and was previously approved by the Internal Review Board of the Ethical and Drug Committee at the University Hospital in Split (reference: 003-08/23-03/0015, approval number: 2181-198-03-04-23-0073). For this study, thirty paraffin-embedded blocks of post-mortem fetal kidney tissue, ranging from the 15th to the 41st gestational week (Table 1), were used. Gestational age was determined based on crown-rump length and menstrual data, while renal pathology classification relied on standard histopathological examination and gross morphological assessment. The renal tissue was fixed in 4% paraformaldehyde with adjusted fixation times, dissected in blocks, and processed through an alcohol series before being embedded in paraffin. The tissue was serially sectioned on a microtome at a 5 µm thickness. Hematoxylin and eosin (H&E) staining was performed using a standardized procedure on every 10th section to ensure appropriate tissue preservation. Normal fetal kidney development stages and abnormal findings in the kidneys damaged by CAKUT were examined using light microscopy.

### 4.2. Hematoxylin and Eosin (H&E) Staining

Mayer’s Hematoxylin (Sigma-Aldrich, Taufkirchen, Germany) was applied to the tissues for 10 min, and then they were submerged in running water for 10 min. The slides were stained cytoplasmically by immersing them in Phloxin B (Merck Millipore, Burlington, MA, USA) and Eosin Y (yellowish) (Merck Millipore) in a ratio 1:2 for a duration of 1 min. The samples were mounted in Canada-balsam (Claro-Prom, Zagreb, Croatia) after being dehydrated in progressively graded ethanol and xylene. H&E staining was used for histological analysis of tissue structures.

### 4.3. Immunofluorescence (IF) on Postmortem Human Prenatal Renal Tissue

Before proceeding with the IF protocol, a standardized procedure for deparaffinizing sections was performed using xylol and alcohol series. Antigen retrieval was performed by boiling the sections in 0.01 M citrate buffer (pH 6.0). After three washes in PBS, a blocking solution (ab64226, Abcam, Cambridge, UK) was applied to the sections for 20 min. The blocking solution was replaced with primary antibodies diluted in PBS and incubated overnight in a humid chamber. After incubation, the sections were washed in PBS, and appropriate secondary antibodies were added and incubated for 1 h at RT. Following three washes in PBS, the sections were incubated for 2 min with DAPI (4′,6-diamidino-2-phenylindole) (Abcam, Cambridge, UK), a fluorescent dye that specifically binds to DNA and covered with coverslips using a mounting medium (Immuno-Mount, Thermo Shandon, Pittsburgh, PA, USA). Exclusion of primary antibodies during the staining process served as a control to ensure the specificity of staining. Antibodies used in this study are summarized in Table 2.

### 4.4. Data Collection & Image Analysis

Imaging was performed utilizing an epifluorescence microscope (Olympus BX51, Tokyo, Japan) and photographed using a Nikon DS-Ri2 camera (Nikon Corporation, Tokyo, Japan) with NIS-Elements F software (version 5.22. 00). ITGA8 and VANGL2 were examined in at least 10 non-overlapping, representative regions of the fetal kidney cortex. All figures were taken at 40× magnification with a consistent exposure duration. Positive staining for ITGA8 and VANGL2 appeared as diffuse or punctuated green staining.

Image analysis was conducted using ImageJ software (version 1.54, National Institutes of Health, Bethesda, MD, USA) as previously described [32,33]. The fluorescence percentage area was determined using the “analyze particles” option. Initially, fluorescence leakage was minimized by subtracting the red countersignal from the green fluorescence. Images were duplicated, and the median filter with a radius of 7.0 pixels was applied to one of the images. The positive signal was isolated by subtracting the filtered images from the unfiltered images. Subsequently, the resulting images were turned into 8-bit images and underwent adjustment using the threshold method (specifically, the triangle thresholding algorithm). To address potential inter-observer differences, three expert histologists independently analyzed the captured microphotographs, establishing background thresholds using negative control images. Interrater agreement was confirmed through interclass correlation analysis, yielding a coefficient greater than 0.8, indicating excellent agreement [34].

### 4.5. Statistical Analysis

All calculations were conducted in Excel v2403 (Microsoft Corporation, Redmond, WA, USA). Statistical analyses were performed using 8.4.3 GraphPad Software (GraphPad Software, La Jolla, CA, USA), with significance set at *p* < 0.05. The normality of the distribution of the data was tested using the Shapiro–Wilk test. The data showed normal distribution. Group comparisons for immunoexpression were conducted using one-way ANOVA, followed by post hoc Tukey’s test.

Linear and nonlinear regression modeling techniques were employed to analyze the expression dynamics and trends of the observed proteins across the four developmental phases of healthy control kidneys. This involved calculating the average area percentage values of the kidney cortex across samples representing different developmental phases. Coefficients, defined as point estimates ± standard error, were used in trend description models, with the coefficient of determination (R^2^) serving as a metric to assess goodness of fit. The slope (β) of a linear regression line was utilized to describe linear trends.

Graphs were produced using GraphPad Prism 8.4.3, while the figures were assembled using Adobe Photoshop software v9.0 (CS2). Background reduction and contrast enhancement were applied to the microphotographs to enhance their presentation.

## 5. Conclusions

Our findings hold significance as they reveal variations in the immunoexpression of ITGA8 and VANGL2 throughout typical human kidney development and in renal disorders. Further observational investigations involving human samples and experimental inquiries utilizing animal models are necessary to deepen our comprehension of these proteins’ roles in both normal kidney development and CAKUT.

## Figures and Tables

**Table 1:** Samples of human fetal kidneys (*n* = 30) analyzed in this study.

Groups	Developmental Phase	Renal and Associated Pathology	Gestational Week	Number of Kidney Samples
Healthy kidney (CTRL)	Phase 2	N/A	15	1
		N/A	16	1
		N/A	17	1
		N/A	18	2
		N/A	21	1
	Phase 3	N/A	23	2
		N/A	28	3
		N/A	29	1
		N/A	32	1
		N/A	35	1
	Phase 4	N/A	37	1
		N/A	38	1
Horseshoe kidney (HK)	Ren concreatus arcuatus, cystae multiplices corticales		22	1
	Ren concreatus arcuatus, tetras Fallot		26	1
	Syndroma Edwards, Ren arcuatus		30–31	1
	Syndroma Edwards, Ren arcuatus		34	1
Dysplastic kidneys (DYS)	Megaureter lateris dextri, Dysplasia renis		21	1
	Dysplasia multicystica renis dextri		27	1
	Cystes parvae focales			
	Renes dysplastici cystici, Syndroma Potter		35	1
	Agenesis renis dextri et dysplasia renis sinistri cum ureter duplex		37	1
	Dysplasia hypoplastica, renis bilateralis, syndroma Down, syndroma Potter		38	1
Hypoplastic kidneys (HYP)	Hypoplasia renis lateris sinistri		37	1
	Hypoplasia renis		38	1
Duplex kidneys (DKs)	Ureter duplex lateris dextri		24	1
	Ureter duplex lateris sinistri		30	1
	Pyelon et ureter duplex bilateralis		41	1

**Table 2:** Antibodies used in this study.

Antibodies		Catalog Number	Host	Dilution	Source
Primary	Anti-ITGA8 antibody	AMAb91468	Mouse	1:200	Sigma-Aldrich (St. Louis, MO, USA)
	Anti-VANGL2 antibody	AF4815	Sheep	1:50	R&D Systems (Minneapolis, MN, USA)
Secondary	Anti-Mouse IgG, Alexa Fluor^®^ 488	711-545-150	Donkey	1:300	Jackson Immuno Research Laboratories, Inc. (Baltimore, PA, USA)
	Anti-Goat IgG (H + L), Alexa Fluor^®^ 488	705-545-003	Donkey	1:300	Jackson Immuno Research Laboratories, Inc. (Baltimore, PA, USA)

## Data Availability

All data and materials are available upon request.
